# Status after Hospital Discharge: An Observational Study of the Progression of Patients’ Mental Health Symptoms Six Weeks after Hospital Discharge

**DOI:** 10.3390/jcm12247559

**Published:** 2023-12-07

**Authors:** Wanying Mao, Reham Shalaby, Ernest Owusu, Hossam Elgendy, Nermin Shalaby, Belinda Agyapong, Angel Nichols, Ejemai Eboreime, Nnamdi Nkire, Vincent I. O. Agyapong

**Affiliations:** 1 Department of Psychiatry, University of Alberta, Edmonton, AB T6G 2R3, Canadannamdi.nkire@albertahealthservices.ca (N.N.); 2 Queen Elizabeth II Hospital, Alberta Health Services, Grande Prairie, AB T5J 3E4, Canada; 3 Department of Psychiatry, Faculty of Medicine, Dalhousie University, 5909 Veterans Memorial Lane, 8th Floor, Abbie J. Lane Memorial Building, QEII Health Sciences Centre, Halifax, NS B3H 2E2, Canada; ejemai.eboreime@dal.ca

**Keywords:** anxiety, depression, well-being, inpatient, discharge, mental health

## Abstract

(1) Background: Transitioning from mental health inpatient care to community care is often a vulnerable time in the treatment process where additional risks and anxiety may arise. We collected data for this study as part of a pragmatic cluster-randomized, longitudinal approach in Alberta. As the first phase of the ongoing innovative supportive program, this paper assessed the progression of mental health symptoms in patients six weeks after hospital discharge. Factors that may contribute to the presence or absence of anxiety and depression symptoms, as well as well-being, following return to the community were investigated. This provides evidence and baseline data for future phases of the project. (2) Methods: An observational study design was adopted for this study. Data on a variety of sociodemographic and clinical factors were collected at discharge and six weeks after via REDCap. Anxiety, depression, and well-being symptoms were assessed using the Generalized Anxiety Disorder (GAD-7) questionnaire, the Patient Health Questionnaire-9 (PHQ-9), and the World Health Organization-Five Well-Being Index (WHO-5), respectively. Descriptive, chi-square, independent *t*-tests, and multivariate regression analyses were conducted. (3) Result: The survey was completed by 88 out of 306 participants (28.8% response rate). The chi-square/Fisher exact test and independent *t*-test revealed no significant change in the mental health conditions from baseline to six weeks after discharge. It was found that the only significant factor predicting symptoms six weeks after discharge from inpatient treatment was the baseline symptoms in all three logistic regression models. It was four times more likely for those who experienced anxiety and depression at baseline to experience anxiety and depression symptoms six weeks after discharge (OR = 4.27; 95% CI: 1.38–13.20) (OR = 4.04; 95% CI: 1.25–13.05). Those with poor baseline well-being were almost 12 times more likely to experience poor well-being six weeks after discharge (OR = 11.75; 95% CI: 3.21–42.99). (4) Conclusions: Study results found no significant change in mental health conditions in the short term following hospital discharge. It is essential that researchers and policymakers collaborate in order to implement effective interventions to support and maintain the mental health conditions of patients following discharge.

## 1. Introduction

The transition from psychiatric hospital treatment to outpatient care can be challenging for many patients [1,2]. It poses substantial risks of serious and even life-threatening adverse outcomes. Examples include premature treatment disengagement [3,4], which subsequently increases the risk of relapse and hospital readmissions [5,6,7,8,9], homelessness [10,11], violent conduct [10,11], involvement with the criminal justice system [12,13], and all-cause mortality, including suicide [1,2,14,15,16]. In light of the frequent occurrence of failed transitions from inpatient to outpatient mental health care, these risks are particularly concerning [1].

Readmission is one of the most well-documented occurrences among individuals with mental health issues throughout the world, ranging from 33% at 3 months post-discharge to 41% at 1 year [2]. Psychiatric disorders are associated with the highest readmission rates among all hospitalized patients in Canada. It has been reported that, for every nine discharges from a mental health inpatient unit in Canada, one patient will be readmitted within 30 days [9,17]. For decades, deinstitutionalization and a transition to community-based mental health care has been an area of focus [18,19,20]; nevertheless, hospital readmissions continue to be a concern. The unmet need for psychological treatment along with a lack of human resources is one of the major reasons for high 30-day readmission rates in psychiatric departments [9]. It is, however, important to note that the variety of challenges that are present at this sensitive time in the patients’ recovery journey after inpatient treatment go far beyond what can be measured solely by the number of readmissions or suicides committed.

There have been numerous follow-up interventions and services developed and tested throughout the world in order to improve and maintain the recovery of patients after discharge. The intent of certain interventions is to reach a specific group, for example, reducing the likelihood of homelessness following discharge [21,22]. Some interventions, such as medication management, address a particular source of health risks following discharge [23,24], whilst others are concerned with coordinating care between several agencies, in a broader sense [25,26]. In Alberta, Canada, through the provision of daily supportive text messages (Text4Support) and Mental Health Peer Support, an innovative supportive program has been developed to fill the gaps in the availability of care and support at the community level for individuals discharged from inpatient psychiatry units and referred to community mental health services for further follow-up [9]. The goal is to reduce the psychological treatment and support gap for those who have been discharged from acute care and have been waiting a long time for mental health and psychiatric treatment from Alberta mental health (AMH) services [9].

As the first phase of this innovative program, the aim of this study was to assess the progression of mental health symptoms six weeks after discharge among a cohort of patients recruited for a clinical trial prior to receiving any intervention. This would enable a better understanding of the challenges associated with the transition from inpatient to outpatient treatment in the mental health field. In view of the difficulties that patients face when transitioning from hospital to community, we hypothesized that their mental health status would deteriorate six weeks after discharge. Three measures (GAD-7, PHQ-9, and WHO-5) were adapted to assess the progression of mental health symptoms in terms of anxiety, depression, and well-being. Additionally, we investigated the potential factors that may have contributed to the likelihood of anxiety, depression, and poor well-being after returning to the community, which provided evidence and baseline data for future phases of the program in identifying potential issues associated with transferring from acute mental health inpatient care to community care.

## 2. Methodology and Materials

### 2.1. Study Design and Data Collection

This study was conducted in Alberta, Canada. According to the Alberta population estimates released by the Government of Alberta on 1 July 2023, the province of Alberta has a population of 4,695,290. The data in this study was collected as a part of a pragmatic stepped-wedge, cluster-randomized, longitudinal approach employed to provide supportive text messages (Text4Support) and peer support services (PSS) that was launched in March 2022, which aims to improve care and support for people discharged from inpatient psychiatry units and referred to community mental health services. Detailed information about this study can be found in the study protocol [9].

In this sub-study, patients were recruited in the first quartile (Q1) of the first year. Data was collected only from the control group in the randomized trial who received only usual follow-up care from Alberta Health Services and primary care providers following discharge without any additional intervention between 8 March and 31 May 2022. Patients were only provided with a baseline survey prior to discharge and a follow-up survey six weeks after enrollment.

Through this project, patients were recruited from eleven acute care hospitals located across four cities in Alberta. After signing a paper-based consent form, all study participants completed the baseline online survey on a tablet through a self-administered online questionnaire via REDCap—a secure browser-based application for building and managing online surveys and translational research databases—with assistance from a member of the research team. Essential sociodemographic factors (e.g., age, sex, ethnicity, relationship status, etc.) and clinical factors (e.g., clinical history of depression, anxiety, and well-being) were collected. Six weeks following their discharge, they received a text message with a link to the follow-up survey. A reminder text message was sent two days after the first follow-up message in order to maximize the response rate. As the primary means of identifying service users, phone numbers are used to track responses over the course of follow-up.

### 2.2. Ethics Statement

This study was approved by the Health Research Ethics Board of the University of Alberta (Ref # Pro00111459). Additional operational approval was obtained from the regional health authority. Signed written informed consent was obtained from participants prior to inclusion into the study. Ethical approval was also obtained for verbal consent in interviews and implied consent in electronic surveys.

### 2.3. Inclusion & Exclusion Criteria

Inclusion criteria for this study included participants with a mental health disorder diagnosis, about to be discharged from an inpatient psychiatry unit, at least 18 years old, who own a mobile device with an active phone number. The participants were able to receive text messages, read English texts, and provided consent forms. In case of out-of-town travel during the 12-month follow-up period, patients were not eligible and were excluded from this study. The research team was assisted in recruiting participants with the assistance and support of physicians, nurses, and unit managers at various sites. The cohort of this study were the participants of the first phase of the innovative project who did not receive any interventions (control group).

### 2.4. Sample Size Calculation

With a projection that the effect size for the reduction in mean GAD-7, PHQ-9, and WHO-5 scores at six weeks from baseline would be 0.5, and with a population variance of 1.0 for each scale mean score, a two-sided significance level α = 0.05, and a power of 90% (β = 0.1), using an online script [27] we estimated that the sample size needed to assess the effects of the six-week transition from inpatient psychiatric care to community care on the outcome variables would be 44.

### 2.5. Outcome Measures

#### 2.5.1. GAD-7

The Generalized Anxiety Disorder (GAD-7) questionnaire was used to assess anxiety symptoms among respondents. Seven self-reported items are used to assess the severity of symptoms associated with GAD over the past two weeks. Ratings are based on a 4-point Likert scale. The responses to each question were as follows: Not at all sure (0), Several days (1), Over half the days (2), and nearly every day (3). The scores ranged from 0 to 21, with higher scores indicative of more severe symptoms of GAD [28]. Research and clinical practice have recommended the GAD-7 scale as the most valid tool to assess the severity of GAD symptoms [28,29]. With a threshold score of 10, the GAD-7 has a sensitivity of 89% and a specificity of 82% for generalized anxiety disorder [28]. The GAD-7 also showed good test–retest reliability (intraclass correlation = 0.83), as well as excellent internal consistency (Cronbach α = 0.92) [29,30].

#### 2.5.2. PHQ-9

The Patient Health Questionnaire-9 (PHQ-9) is a self-report measure based on the 9 DSM-IV criteria for major depression. Symptoms experienced by patients during the two weeks prior to answering questionnaires are considered. Scores range from never (0) to several days (1), more than half of days (2), and 3 (nearly every day), with every item ranging from 0 to 27 [31,32]. Major depression can be diagnosed using the PHQ-9 screening tool, with a recommended cut-off score of ten or greater. Scores of 5, 10, 15, and 20 represented mild, moderate, moderately severe, and severe depression, respectively [31]. The Cronbach’s alpha for the PHQ-9 is 0.851, indicating good internal consistency. As well as this, moderate to strong correlations were seen with WHO-5, HADS-depression, HADS-anxiety, and GAD-7 measures, confirming convergent validity. Using a cut-off of ≥10, the PHQ-9 demonstrated good sensitivity (0.88) and specificity (0.88) [31,32,33].

#### 2.5.3. WHO-5

The World Health Organization-Five Well-Being Index (WHO-5) is a self-reported measure of mental well-being developed by the World Health Organization. It contains five positively worded items: “I have felt cheerful and in good spirits”; “I have felt calm and relaxed”; “I have felt active and vigorous”; “I woke up feeling fresh and rested”; and “My daily life has been filled with things that interest me” [34]. A 6-point Likert scale is used to assess the frequency with which the positive feelings were present in the last two weeks, ranging from 0 (not present) to 5 (constantly present). The raw scores are transformed into a score from 0 (worst thinkable well-being) to 100 (best thinkable well-being). Having a score <50 indicates poor emotional well-being and requires further evaluation [34]. The WHO-5 demonstrated satisfactory internal consistency reliability (α = 0.90) and convergent validity with the PHQ-9 (r = −0.73, *p* < 0.001). Several studies have shown that it has a sensitivity of 93% and a specificity of 83% for identifying depression [34,35].

### 2.6. Statistical Analysis

Statistical analysis was performed using SPSS Version 25 [36]. Descriptive analysis was run initially on the sociodemographic characteristics and presented in terms of frequency and percentage. A chi-square analysis or Fisher exact analysis (for small sample sizes) was conducted to compare the prevalence of anxiety, depression, and poor well-being as assessed by three measures (GAD-7, PHQ-9, WHO-5) at baseline and six weeks following discharge. Using paired sample *t*-test, a comparison was performed between the mean scores of each variable at baseline and six weeks after leaving hospital treatment to evaluate the severity of mental health symptoms under study, including anxiety and depression, as well as well-being.

Among the outcomes of our study, we were interested in examining potential predictors of anxiety, depression, and poor well-being. Therefore, three multivariable binomial logistic regression models were used to identify significant variables, including social demographic characteristics, such as age, gender, ethnicity, educational level, housing status, and current employment status, as well as the related baseline clinical characteristics. For example, baseline depression levels were included when predicting moderate-to-severe depression 6 weeks after. Confidence intervals and odds ratios (ORs) were used to determine the predictor variables. Missing data were not imputed, and only completed responses were reported.

## 3. Results

### 3.1. Demographic Information of the Respondents

A total of 334 service users were contacted for the study, of whom 306 consented and signed up for the project between 8 March and 31 May 2022 and provided baseline survey data. In addition to 11 declining and 17 not meeting the inclusion criteria, 17 individuals were under the age of 18 and did not possess cell phones. As part of the randomized trial, all study participants were assigned to the control group according to a stepped-wedge design. Between 19 April and 12 July 2022, 144 participants attempted the 6-week survey. A total of 37 respondents provided incomplete responses which prevented analysis of the data, 19 used non-valid phone numbers (non-trackable cases), and 88 completed both baseline and six-week surveys, providing valid phone numbers, resulting in an effective response rate of 88/306 = 28.8%.

Descriptive demographic- and clinical-related responses were collected from the participants (N = 88). As shown in Table 1, most of the participants were aged between 26–40 years old (38.6%), females (62.5%), employed (38.6%), Caucasians (68.2%), received postsecondary education (48.9%), single (61.4%), unemployed (45.4%), and were living in rented accommodation (37.5%). In terms of clinical history, most of our participants reported having depression and anxiety diagnoses (38.6%).

### 3.2. Association Analysis Using Chi-Square/Fisher Exact Test

Using a chi-square test, the association between participants’ mental health status at discharge and that six weeks after discharge was examined (Table 2). At discharge, 42 (47.7%) had moderate-to-severe anxiety. After six weeks back in the community, 37 (42%) experienced moderate-to-severe anxiety (Chi^2^(1) = 0.57, *p* = 0.45). Similarly, at discharge, 55 (62.5%) were found to have moderate-to-severe depression, while 46 patients (52.3%) reported moderate-to-severe depression 6 weeks after leaving inpatient treatment (Chi^2^(1) =1.88, *p* = 0.17). In terms of well-being, 35 (39.8%) were identified as having poor well-being at the time at discharge; six weeks after leaving the hospital, 47 (53.4%) reported having poor well-being (Chi^2^(1) =3.29, *p* = 0.07). These apparent changes, however, with *p*-values < 0.05 were not statistically significant.

### 3.3. Mean Score Changes from Baseline to Six Weeks (Paired Sample t-Test)

To evaluate the change in severity of mental health symptoms between baseline and six weeks after discharge, a paired sample *t*-tests was run with a 95% confidence interval (CI) as indicated in Table 3. The *t*-tests was used to compare the mean scores of three scales (GAD-7, PHQ-9, WHO-5). The results demonstrate that among the 88 participants, there were no significant changes to the mental health conditions in the study based on the results comparing baseline to six-weeks data. The anxiety (M = 8.89, SD = ±6.17) and depression mean score (M = 11.21, SD = ±7.71) after six weeks from discharge decreased when compared to the baseline anxiety (M = 9.49, SD = ±5.57) and depression mean score (M = 12.19, SD = ±6.79). However, this change was not statistically significant for both anxiety (t(87) = 0.98, *p* = 0.33) and depression (t(87) = 1.34, *p* = 0.19) symptoms. Participants’ mean score of well-being six weeks after discharge (M = 51.19, SD = ±25.90) declined when compared to the baseline value (M =53.14, SD = ±24.63); this change was not statistically significant, as well (t(87) = 0.72, *p* = 0.47).

### 3.4. Predictors of Likely Anxiety, Depression, and Wellbing (Logistic Regression)

Three multivariable binomial logistic regression models were used to determine the likely predictors of moderate-to-severe anxiety and depression symptoms as well as poor well-being among the study respondents (see Appendix A (Table A1, Table A2 and Table A3)). Seven demographic and clinical predictor variables were included in all the three models, which are age, gender, ethnicity, education level, current employment status, current housing status, and baseline anxiety/depression/poor well-being scores, respectively.

The logistic regression model of anxiety was statistically significant with χ^2^ (15) = (26.67, *p* ≤ 0.032), suggesting that the model could distinguish respondents with moderate-to-severe anxiety symptoms and those with the lowest level of anxiety six weeks following hospital discharge (see Appendix A (Table A1)). The model explained 26.1% (Cox and Snell R^2^) to 35.2% (Nagelkerke R^2^) of the variance, showing the likelihood that respondents will report symptoms of anxiety and accurately identifying 72.7% of the cases. As indicated, only one variable, “*baseline likely anxiety*”, was significant in predicting moderate-to-severe anxiety symptoms among participants six weeks after discharge in the model (*p* ≤ 0.05). In comparison with those without baseline anxiety symptoms, those who reported experiencing moderate-to-severe anxiety at baseline were four times more likely to experience moderate-to-severe anxiety symptoms six weeks after discharge (OR = 4.27; 95% CI: 1.38–13.20).

A logistic regression model was used to predict possible risk factors of depression symptoms among study participants, six weeks after discharge (see Appendix A (Table A2)). Statistically, the entire model was significant, χ^2^ (15) = (28.78, *p* ≤ 0.017), demonstrating that the model was able to distinguish between respondents who had moderate-to-severe depression and those who did not 6 weeks after hospital discharge. As a consequence, the model explained 27.9% (Cox and Snell R^2^) and 37.2% (Nagelkerke R^2^) of the variance, indicating the likelihood that respondents would report symptoms of depression and accurately identifying 68.2% of the cases of depression. In line with the model of anxiety, the “*baseline depression mean score*” was the only significant predictor of depression six weeks after discharge (*p* ≤ 0.05). There is a fourfold increase in the likelihood of participants with baseline depression symptoms experiencing moderate-to-severe depression six weeks after discharge in comparison with those without baseline depression indications (OR = 4.04; 95% CI: 1.25–13.05).

Regarding the logistic regression model of well-being (see Appendix A (Table A3)), the entire model was statistically significant, χ^2^ (19) = (34.06, *p* ≤ 0.018), demonstrating that it could differentiate between respondents who had a good well-being 6 weeks after discharge and those who did not. The model explained 32.1% (Cox and Snell R^2^) to 42.9% (Nagelkerke R^2^) of the variance, indicating that respondents are likely to present with poor well-being and correctly identifying 78.4% of cases. According to the model, the “*baseline poor well-being*” was the only significant variable in predicting poor well-being among participants six weeks following discharge (*p* < 0.001). In comparison with patients with good baseline well-being, those of poor baseline well-being were almost 12 times more likely to experience poor well-being six weeks after discharge (OR = 11.75; 95% CI: 3.21–42.99).

## 4. Discussion

Our hypothesis was not supported by the study results as there was no significant reduction or even change in mental health status between baseline and six weeks, which contradicts the existing findings that the patients’ mental health would deteriorate after discharge [9,17,37,38]. In an Australian study, 135 patients who had been discharged from an acute psychiatric ward were found to have more psychiatric symptoms and to have more disturbed behavior six months after discharge [37]. It was found in a Brazilian study conducted in São Paulo that patients with psychotic and bipolar disorders showed deterioration within one year after discharge, resulting in a high rate of readmission [37,38]. As Olfson et al. observed, individuals with schizophrenia are particularly susceptible to worsening conditions after discharge, which can result in homelessness and rehospitalization [37,38]. There may be several reasons why our study did not reach a similar result, such as the small sample size, which undermines both the internal and external validity of the study and makes extrapolation more difficult [39]. Considering that most of our participants are white, racial disparities may also play a role. Studies have documented that racial disparities persist after adjustment for sociodemographic and diagnostic confounders, and they appear to persist regardless of gender, socioeconomic status, or psychiatric diagnosis [40]. Research has repeatedly shown that ethnic minority groups are more likely to suffer from severe and long-term mental health problems [40,41,42]. It is also possible that six weeks does not provide sufficient time to observe significant changes in participants’ mental health.

### 4.1. Anxiety

Among the seven variables evaluated in this study, only the “*baseline anxiety mean score*” factor was significant in predicting moderate-to-severe anxiety symptoms six weeks following discharge. As compared to those without baseline anxiety symptoms, those with baseline anxiety symptoms were four times more likely to experience moderate-to-severe anxiety symptoms six weeks later after discharge. Similar results were found in a previous study that assessed patterns and predictors of anxiety symptoms over time following a traumatic wildfire in Fort McMurray, Canada [30]. A longitudinal study identified that 23.5% of 429 patients with a history of anxiety disorders but without a current anxiety disorder at baseline showed recurrent anxiety symptoms within 2 years [43], and a similar result was found in another sample from the same cohort [44]. The rate of relapse remains high even when psychological treatment has been successfully completed. The results of a meta-analysis of nine studies conducted on relapse after cognitive behavioral therapy (CBT) for anxiety disorders (N = 532 patients) indicate that, on average, 23.8% of patients experienced relapse after undergoing CBT [45].

Predictors of recursive anxiety are mostly investigated in clinical samples. Comorbid depression and anxiety disorders, as well as higher levels of dysfunction, were found to predict recurrence of previous anxiety disorders [46,47,48,49,50]. In addition, it was found that vulnerability characteristics such as neuroticism, high anxiety sensitivity, and parental substance abuse were also risk factors [49,50,51,52]. Among these studies, only two included multivariate analyses of sociodemographic, clinical, and vulnerability characteristics [43,49]. A multivariate analysis of the data from both studies identified ‘anxiety sensitivity’ (having a fearful reaction to anxiety symptoms) as a risk factor of recurrence, in addition to higher levels of dysfunction. It was noted by Taylor et al. that being single, smoking, and benzodiazepines treatment were also associated with the occurrence of recurrences [49].

### 4.2. Major Depression Disorder

As was the case of anxiety, results found in our study related to depression showed that the variable “*baseline depression mean score*” was the only significant predictor of depression six weeks after discharge (*p* ≤ 0.05). When compared with those without baseline depression indicators, participants with baseline depression symptoms have a fourfold higher chance of experiencing moderate-to-severe depression six weeks after discharge. Our results are consistent with prior research that aimed to assess the patterns and predictors of depressive symptom trajectories over time [32,53,54,55,56,57,58].

Major depression disorder has been demonstrated to be highly recurrent in many publications, with at least 50% of those who recover from their first episode experiencing another episode within a lifetime, and approximately 80% of those with two episodes experiencing another recurrence [57,59,60]. A person with a history of depression is likely to suffer five to nine separate episodes of depression during their lifetime [57]. A significant amount of research suggests that early onset of depression [61,62], severe early episodes [63], comorbidity psychopathology [64,65], family history of psychopathology [66,67], and stressful life events, both as a child and particularly as an adult, contribute to depression recurrence [68,69,70]. While demographic variables such as gender, marital status, or socioeconomic status may play a crucial role in the onset of depression, they are not thought to be significant predictors of the risk of recurrence [57]. This aligns with our findings, where socio-demographic characteristics failed to significantly predict the likelihood of depression six weeks following hospital discharge.

### 4.3. Well-Being

Among the variables identified in the logistic regression model regarding the well-being, only “*baseline poor well-being*” had a significant effect on predicting poor well-being among participants six weeks following discharge. Comparatively to patients with high baseline well-being scores at discharge, those with low baseline well-being scores had a 12-times greater likelihood of experiencing poor well-being six weeks following discharge.

Unfortunately, a lack of literature has been found regarding the trajectory of the well-being score over time, which may motivate future research examining how the well-being of patients has been impacted over time under different circumstances. In spite of this, numerous studies have provided sufficient evidence concerning the risk factors for poor well-being and quality of life. Genetics, personality, and demographic factors all affect quality of life and well-being on an individual level. Positive emotions, for example, are heritable to a certain extent, suggesting that emotions such as happiness and sadness have a genetic setting point [71,72,73]. It has been shown that optimism, extroversion, and self-esteem are significant personality factors associated with both well-being and quality of life [74,75]. In terms of determining the well-being of an individual, genetic factors and personality factors are closely related and can interact with each other. Well-being has also been shown to be related to age and gender. The levels of well-being of men and women are generally comparable, but this pattern has changed over time as well as with age [76,77]. A U-shaped pattern of well-being is observed with age, with younger and older adults experiencing greater well-being than middle-aged adults [78]. Additionally, longitudinal studies show that well-being is sensitive to life events such as unemployment and marital transitions. Several studies have demonstrated that unemployment negatively impacts both the short- and long-term well-being of an individual [78,79,80]. As for marriage, although there are substantial differences between individuals, having supportive relationships is one of the strongest predictors of a sense of good well-being [79]. This study, however, found no significant effect of such characteristics, including gender, marital status, employment status, or age, on having a poor well-being after hospital discharge; rather, the perceived poor well-being at baseline was the strongest predictor.

While this was not revealed by our study, it is not surprising to imagine that after an inpatient stay and improvement in mental health symptoms, patients with mental illness may have concerns and dilemmas about life after discharge [81]. With numerous challenges ahead, these individuals may feel frustrated and helpless as the future becomes uncertain. Studies identified challenges from the following examples: (1) problems relating to medication management and keeping concordance; (2) increased risk of self-harm as well as others (particularly family members); (3) a lack of information sharing among services resulting in both gaps and duplication in services; and (4) a poorer mental health due to multiple, often difficult, transitions that can cause distress [70,81,82]. Several factors have also been identified as contributing to the poor post discharge situation in the literature including a lack of insight, an absence of social support, a poor doctor–patient relationship, wrong conclusions about whether certain medications were necessary, and a lack of awareness about the illness [1,2,14,15,16].

It cannot be stressed enough that continuity of care following discharge from the hospital is an essential element of high-quality patient care. To provide highly reliable care, there must be close cooperation between health care providers across organizational boundaries, resulting in the formation of an interdisciplinary team. Additionally, innovative supportive interventions, such as evidence-based supportive text messaging and mental health peer-support programs, should be promoted vigorously to assist recovery and to increase access to high quality community mental healthcare, thereby reducing acute care and inpatient hospitalizations.

## 5. Limitations

This study is limited by a relatively small sample size. The results may need to be replicated in larger studies. Since a majority of the participants in this study were white/Caucasian, it may limit generalization to non-white/Caucasian populations. The low response rate (28.8%) is another limitation of this study. Although we reached our target sample size and a low response rate does not necessarily affect the validity of the collected data, in order to maximize validity, it is still necessary to test for non-response effects and make corrections to the original data for future studies. Furthermore, the change was examined over a relatively short period of time, over which there may still be changes that have yet to be manifested. There may be a need for further research to conduct similar comparisons over a longer period. Also, the result might be affected by health utilization parameters including prior hospitalizations, emergency visits, and follow up visits after discharge, etc. Finally, the use of self-reported measuring scales in this study, including GAD, PHQ, and WHO-5 Well-being Index, may leave room for bias and adversely impact the objectivity of the information provided by the respondents.

## 6. Conclusions

This study identified the prevalence and severity of anxiety, depression, and well-being among participants at the time of hospital discharge and 6 weeks following their discharge from eleven acute care hospitals and day hospitals located across four cities in Alberta, Canada, resulting in increased clinical and research evidence in the field of inpatient and discharge care. The results did not support our hypothesis since there was not a significant reduction or change in mental health symptoms from baseline to six weeks after, which suggests that the relationship between variables might have been due to chance. Symptoms at baseline were identified as the only significant predictors of symptoms after discharge.

After discharge from an inpatient mental health facility, there is often a chaotic and emotionally charged period that is characterized by several risk factors. To address some of these issues, several interventions have been developed internationally, with varying degrees of success. By improving the homogeneity of outcome reporting and applying the theory of change to future research, a better comparison of interventions may be possible. Additionally, policymakers should collaborate closely with scientists to advance evidence-based policies and practices aimed at improving mental health, particularly discharge services.

## Figures and Tables

**Table 1:** Baseline distribution of sociodemographic and clinical characteristics.

Variables	N = 88	%
**Gender**		
Male	28	31.8
Female	55	62.5
Other	5	5.7
**Age (Years)**		
≤25	21	23.9
26–40	34	38.6
41–60	24	27.3
>60	9	10.2
**Ethnicity**		
White	60	68.2
Indigenous	4	4.5
African	7	8.0
Asian	12	13.6
Other	5	5.7
**Educational level**		
Less than high school	6	6.8
High school	39	44.3
Postsecondary education	43	48.9
**Relationship status**		
Single	54	61.4
Separated/divorced	11	12.5
Partnered/married	23	26.1
**Employment status**		
Employed	34	38.6
Unemployed	40	45.4
Student	7	8.0
Retired	7	8.0
**Housing status**		
Own home	25	28.4
Rented accommodation	33	37.5
Live with family or friend	30	34.1
**Primary Mental Health Diagnosis**		
Depression/anxiety	34	38.6
Bipolar disorder	18	20.5
Psychosis	14	15.9
Alcohol, drug use/abuse	7	8.0
Other	15	17.0

**Table 2:** Change in the prevalence of categorical scales, six weeks after hospital discharge.

Measures	Baseline n (%)	Six Weeks after Discharge n (%)	Total	Chi Square/ Fisher Exact	*p*-Value
**GAD-7** ^1^					
At most low anxiety	46(52.3%)	51 (58.0%)	97 (55.1%)	0.574	0.449
Moderate-to-severe anxiety	42(47.7%)	37 (42.0%)	79 (44.9%)		
**PHQ-9** ^2^					
At most mild MDD	33 (37.5%)	42 (47.7%)	75 (42.6%)	1.882	0.170
Moderate-to-severe MDD	55 (62.5%)	46 (52.3%)	101 (57.4%)		
**WHO-5** ^3^					
Good well-being	53 (60.2%)	41 (46.6%)	94 (53.4%)	3.288	0.070
Poor well-being	35 (39.8%)	47 (53.4%)	82 (46.6%)		

^1^ GAD-7: Using the threshold score of 10. A score 10 or greater represents moderate-to-severe anxiety. ^2^ PHQ-9: Using the threshold score of 10. A score 10 or greater represents moderate-to-severe depression. ^3^ WHO-5: Using the threshold score of 50. A score 50 or greater represents good well-being.

**Table 3:** Change in mean scores of clinical characteristics six weeks after hospital discharge.

Measure	Responses, n	Scores			Mean Difference (95% CI)	*p*-Value	*t*-Value
		Baseline Score, Mean (SD)	Six-Week Score, Mean (SD)	Change from Baseline, %			
**GAD-7** ^1^	88	9.49 (5.57)	8.89 (6.17)	−6.35%	(−0.62–1.83)	0.331	0.977
**PHQ-9** ^2^	88	12.19 (6.79)	11.21 (7.71)	−8.12%	(−0.48–2.46)	0.185	1.337
**WHO-5** ^3^	88	53.14 (24.63)	51.18 (25.90)	−3.68%	(−3.43–7.34)	0.473	0.721

^1^ GAD-7: Using the threshold score of 10. A score 10 or greater represents moderate-to-severe anxiety. ^2^ PHQ-9: Using the threshold score of 10. A score 10 or greater represents moderate-to-severe depression. **^3^** WHO-5: Using the threshold score of 50. A score 50 or greater represents good well-being.

## Data Availability

The data presented in this study are available on request from the corresponding author. The data are not publicly available due to privacy and ethical reasons.

## Appendix A

**Table A1:** Logistic regression predicting likelihood of respondents self-reporting likely anxiety.

		B	S.E.	Wald	df	Sig.	Exp(B)	95% C.I. for EXP(B)	
								Lower	Upper
Age	≤25 y			1.125	3	0.771			
	26–40 y	0.127	0.842	0.023	1	0.880	1.135	0.218	5.917
	41–60 y	0.190	0.902	0.044	1	0.833	1.209	0.206	7.089
	>60	1.121	1.186	0.894	1	0.344	3.069	0.300	31.393
Gender	Male			0.247	2	0.884			
	Female	0.295	0.594	0.247	1	0.619	1.344	0.420	4.303
	Other	38.169	20,341.982	0.000	1	0.999	3772 × 10^16^	0.000	
Ethnicity	Caucasian			3.728	4	0.444			
	Indigenous	−18.895	13,525.265	0.000	1	0.999	0.000	0.000	
	Asian	0.601	0.840	0.512	1	0.474	1.824	0.352	9.453
	African descendants	−0.991	1.211	0.669	1	0.413	0.371	0.035	3.988
	Other	1.648	1.098	2.252	1	0.133	5.195	0.604	44.689
Education level	Less than high school			1.112	2	0.574			
	High school	0.729	1.099	0.440	1	0.507	2.073	0.241	17.861
	Post-secondary education	0.086	1.129	0.006	1	0.939	1.090	0.119	9.960
Housing status	Own home			0.406	2	0.816			
	Rented accommodation	−0.402	0.786	0.262	1	0.609	0.669	0.143	3.123
	Live with family or friends	−0.493	0.824	0.358	1	0.549	0.611	0.121	3.071
Employment status	Unemployed	−0.728	0.622	1.371	1	0.242	0.483	0.143	1.633
GAD-7 score at baseline		1.451	0.576	6.350	1	0.012	4.268		
Constant		−1.279	1.396	0.840	1	0.359	0.278		

**Table A2:** Logistic regression predicting likelihood of respondents self-reporting likely depression.

			B	S.E.	Wald	df	Sig.	Exp(B)	95% C.I. for EXP(B)	
									Lower	Upper
Age	≤25 y				1.266	3	0.737			
	26–40 y		−0.170	0.851	0.040	1	0.841	0.843	0.159	4.468
	41–60 y		0.404	0.906	0.198	1	0.656	1.498	0.253	8.850
	>60		0.844	1.146	0.542	1	0.461	2.325	0.246	21.971
Gender	Male				0.801	2	0.670			
	Female		0.512	0.572	0.801	1	0.371	1.669	0.544	5.121
	Other		38.789	20,133.183	0.000	1	0.998	7014 × 10^16^	0.000	
Ethnicity	Caucasian				2.016	4	0.733			
	Indigenous		−19.277	13,391.057	0.000	1	0.999	0.000	0.000	
	Asian		−0.735	0.837	0.772	1	0.380	0.479	0.093	2.471
	African descendants		0.046	1.036	0.002	1	0.965	1.047	0.137	7.981
	Other		1.170	1.132	1.069	1	0.301	3.224	0.351	29.636
Education level	Less than high school				0.692	2	0.708			
	High school		0.006	1.048	0.000	1	0.995	1.007	0.129	7.855
	Post-secondary education		−0.557	1.118	0.248	1	0.618	0.573	0.064	5.124
Housing status	Own home				2.571	2	0.276			
	Rented accommodation		−0.497	0.793	0.393	1	0.531	0.609	0.129	2.876
	Live with family or friends		−1.324	0.842	2.474	1	0.116	0.266	0.051	1.385
Employment status	Unemployed	0.051		0.615	0.007	1	0.934	1.052	0.315	3.511
PHQ-9 score at baseline			1.396	0.598	5.441	1	0.020	4.038	1.250	13.046
Constant			−0.448	1.359	0.109	1	0.742	0.639		

**Table A3:** Logistic regression predicting likelihood of respondents self-reporting low QOL.

		B	S.E.	Wald	df	Sig.	Exp(B)	95% C.I. for EXP(B)	
								Lower	Upper
Age	≤25 y			1.788	3	0.618			
	26–40 y	0.215	0.893	0.058	1	0.810	1.239	0.215	7.130
	41–60 y	0.716	1.007	0.506	1	0.477	2.046	0.284	14.722
	>60	−0.971	1.477	0.432	1	0.511	0.379	0.021	6.852
Gender	Male			1.032	2	0.597			
	Female	0.139	0.684	0.041	1	0.839	1.149	0.301	4.388
	Other	−1.319	1.539	0.735	1	0.391	0.267	0.013	5.456
Ethnicity	Caucasian			0.400	4	0.982			
	Indigenous	−0.135	1.368	0.010	1	0.922	0.874	0.060	12.762
	Asian	0.347	0.893	0.151	1	0.698	1.414	0.246	8.138
	African descendants	−0.419	1.101	0.145	1	0.704	0.658	0.076	5.695
	Other	−0.114	1.159	0.010	1	0.922	0.892	0.092	8.646
Education level	Less than high school			2.211	2	0.331			
	High school	−1.290	1.205	1.147	1	0.284	0.275	0.026	2.919
	Post-secondary education	−1.918	1.310	2.142	1	0.143	0.147	0.011	1.917
Housing status	Own home			0.512	2	0.774			
	Rented accommodation	−0.210	0.848	0.061	1	0.804	0.810	0.154	4.271
	Live with family or friends	0.354	0.971	0.133	1	0.716	1.424	0.212	9.551
Employment status	Unemployed	−0.215	0.663	0.105	1	0.746	0.807	0.220	2.957
Previous mental health diagnosis	Depression/anxiety			4.170	4	0.383			
	Bipolar disorder	0.598	0.783	0.583	1	0.445	1.818	0.392	8.427
	Psychosis	1.370	0.984	1.938	1	0.164	3.934	0.572	27.050
	Alcohol drug abuse	2.351	1.455	2.613	1	0.106	10.501	0.607	181.792
	Other	0.331	0.855	0.150	1	0.699	1.392	0.261	7.431
Poor well-being (WHO-5) at baseline		2.464	0.662	13.857	1	0.000	11.749	3.211	42.990
Constant		0.095	1.517	0.004	1	0.950	1.100		
